# A Flexible Six-in-One Microsensor Embedded in a Vanadium Redox Flow Battery for Long-Term Monitoring

**DOI:** 10.3390/mi14051032

**Published:** 2023-05-11

**Authors:** Chi-Yuan Lee, Chia-Hung Chen, Yu-Chun Chen, Kai-Siang Fan

**Affiliations:** 1 Department of Mechanical Engineering, Yuan Ze Fuel Cell Center, Yuan Ze University, Taoyuan 32003, Taiwan; 2 HOMYTECH Global Co., Ltd., Taoyuan 33464, Taiwan

**Keywords:** energy storage, VRFB, MEMS, flexible six-in-one microsensor, microscopic monitoring, physical parameter, operating condition

## Abstract

The vanadium redox flow battery (VRFB) can be used as a supporting technology for energy storage corresponding to wind and solar power generation. An aqueous vanadium compound solution can be used repeatedly. As the monomer is large, the flow uniformity of electrolytes in the battery is better, the service life is long, and the safety is better. Hence, large-scale electrical energy storage can be achieved. The instability and discontinuity of renewable energy can then be solved. If the VRFB precipitates in the channel, there will be a strong impact on the flow of vanadium electrolyte, and the channel could even be blocked as a result. The factors which influence its performance and life include electrical conductivity, voltage, current, temperature, electrolyte flow, and channel pressure. This study used micro-electro-mechanical systems (MEMS) technology to develop a flexible six-in-one microsensor which can be embedded in the VRFB for microscopic monitoring. The microsensor can perform real-time and simultaneous long-term monitoring of the physical parameters of VRFB, such as electrical conductivity, temperature, voltage, current, flow, and pressure to keep the VRFB system in the best operating condition.

## 1. Introduction

Low carbon management has become one of the popular research and development issues in the world. Scholars and practitioners around the world have accepted the necessity of realizing zero carbon emission or carbon neutrality at the macroscopic and microscopic levels [1]. With the emergence of more intermittent renewable energy, balancing the hourly supply and the demand of energy and electricity has become crucial to ensure the feasibility of the path [2]. Considering the hourly availability of different renewable technologies, and the hourly variation of electricity requirement, more and more countries and companies aim to realize net zero emissions (NZE) of CO_2_ and support the goal of limiting the global average temperature rise to 1.5 °C by 2100 in the Paris Climate Agreement [3]. In recent years, wind energy, solar energy, and power storage technologies have significantly improved in the aspects of society, politics, technology, and economic feasibility [4]. The proportions of wind energy and solar energy have been increasing gradually, with wind energy and solar energy typically changing seasonally and are restricted by regions. However, renewable energy fuels are still the pillar of NZE of the energy sector [5].

The VRFB has great potential in medium and large energy storage applications [6,7]. The polyelectrolyte membrane is considered as the key component of VRFB devices and has a long cycle life in practical applications [8]. Since vanadium ions are used on both sides of the VRFB, vanadium ions with different valence states are used as anolytes and catholytes, and the capacity regeneration process is simple and cross-contamination can be eliminated [9,10,11,12]. The high-power density VRFB is characterized by its exquisite structure and significantly lower system cost, and its commercial application is increasingly important [13]. To remedy the low energy density of the VRFB, Huang et al. [14] aimed to improve the battery performance, but mainly aimed at the core components of VRFB material, such as electrolytes, electrodes, separator, bipolar plates, and galvanic pile designs. It is particularly important to measure and observe the dynamic response in the battery.

Charvát et al. [15] compared films and found that the thinner and more conductive film had a better performance stability than the thicker and less conductive film. In addition, the reduction of concentration overpotential can extend the charge/discharge time to the voltage limit, so that the battery capacity and power are increased. Therefore, battery energy efficiency always increases with the electrolyte flow. The conductivity is influenced by temperature, vanadium concentration, and sulfuric acid concentration, and the battery conductivity can be improved by either reducing the vanadium concentration or increasing the sulfuric acid concentration [16]. Jienkulsawad et al. [17] observed the concentration overpotential was reduced as the mass transfer of reactant was enhanced by increasing the electrolyte flow velocity. However, the activation and ohmic overpotential do not vary with the electrolyte flow velocity. Tripathi et al. [18] found when the fluid flowed through the pipe, and the friction induced by flow resistance acted on the fluid, there was a pressure drop generated, with the main determinants in fluid flow resistance being the fluid velocity and fluid viscosity through the pipe. Rao et al. [19] found that low temperature led to high electrolyte viscosity, and the permeability of the electrode was therefore enhanced. At temperatures below zero, the pressure drop in the battery and pile increases significantly, so that the pumping loss also increases significantly. A large amount of current is generated when the chemical reaction rate is high and the battery temperature rises, so appropriate cooling is required to avoid safety concerns induced by overheating of the battery. Excess pressures will deteriorate parts in the battery, and therefore the battery efficiency will be reduced as a result. The flow decreases with pressure, the voltage increases as the pressure drop loss decreases, and the polarization loss decreases, meaning the efficiency increases accordingly. The above description shows that the low solubility of the electrolyte will lead to a decrease in the energy density of the VRFB. Increasing the electrolyte flow can therefore improve the energy efficiency and conductivity of the battery. Appropriate cooling measures can avoid safety hazards caused by battery overheating. Excessive stress can reduce battery efficiency and even damage components in the battery.

Therefore, this study used MEMS technology to develop a flexible six-in-one microsensor and embed it in a VRFB for microscopic monitoring. The microsensor performed real-time and simultaneous long-term monitoring of the physical parameters of VRFB, including electrical conductivity, temperature, voltage, current, flow, and pressure, to keep the VRFB system in the best operating condition. Summarizing the above literature, we know the impact of the changes in the amount of each material on the performance and life of the battery. A flexible six-in-one microsensor can therefore be used to measure battery status. It can measure the conductivity, temperature, voltage, current, flow, pressure, and other parameters of the battery, and use these parameters to infer the remaining capacity and the service life of the battery. Additionally, it can provide real-time battery status information to help users better manage their battery usage, thereby extending battery life while also reducing equipment damage and safety risks due to battery failure.

## 2. Development of the Flexible Six-in-One Microsensor

**Figure 1:**
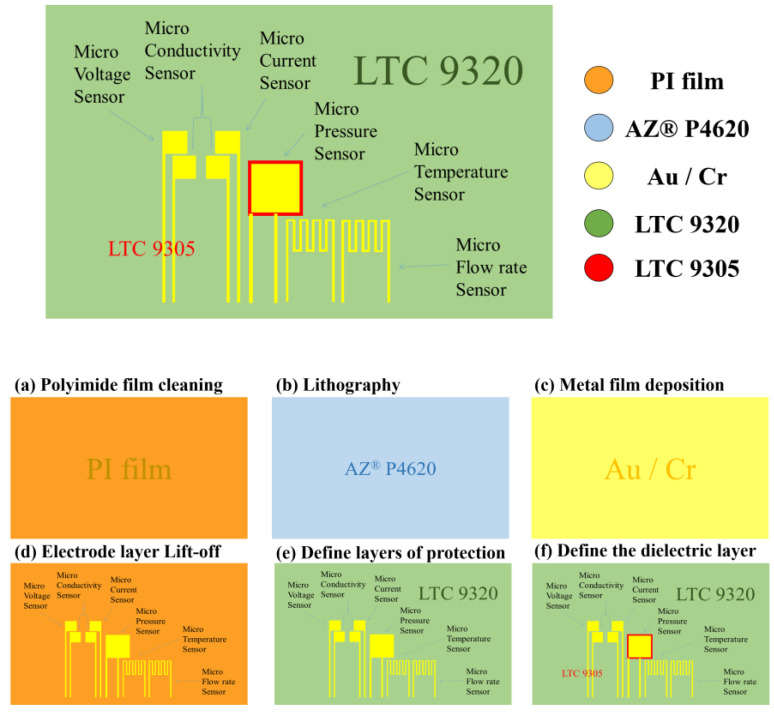
Process diagram of the flexible six-in-one microsensor.

This study used MEMS technology to integrate a flexible six-in-one microsensor for sensing six physical quantities, including electrical conductivity, temperature, voltage, current, flow, and pressure. The schematic diagram of process of the microsensor is shown in Figure 1.

To enhance the adhesive ability of the metal and remove surface dust and grease, the 50 μm thick PI film substrate was washed with two organic solvents, acetone, and methanol, and the surface agent was then washed off with DI water.

The “AZ^®^ P4620” positive photoresist was uniformly spin-coated on the PI substrate, and the mask of the microsensor was then developed on the substrate using lithography. Lithographic development was the critical step, and the quality of development would thereby influence the subsequent overall process.

The electron beam evaporation machine evaporates Cr as the adhesive layer and Au as the sensing electrode layer, respectively. The principle of the electron beam evaporation machine was to use electrons to bombard the target, so that the temperature of the target rises to the melting point, and then evaporates and deposits on the surface of the target. In this study, the evaporation process was carried out at a deposition rate of 0.5 Å/s.

The electrode layer lift-off step uses acetone to remove the original photoresist solution and lifts off the excess metal to leave the metal required for the electrode pattern. In the case that there was still residue present, a cotton swab was utilized to gently wipe it off completely.

Following this, coating LTC 9320 as a dielectric layer and protective layer was conducted on the film substrate. The material selection of the protective layer is extremely important to note. It is necessary to select materials that are insulating, tough, wear-resistant, acid-resistant, have certain temperature resistance, and encompass resistance to electrochemical reactions. The protective layer also has an insulating effect, which can prevent the flexible six-in-one microsensor from contacting the graphite plate and causing a short circuit. The protective layer must be an insulating material, with its purpose being to expose the sensor head of the micro-conductivity, voltage, and current sensor, directly contact with the rib of the flow channel, and expose the signal pin (Pad), to ultimately facilitate subsequent signal transfer and output to the measuring end.

**Figure 2:**
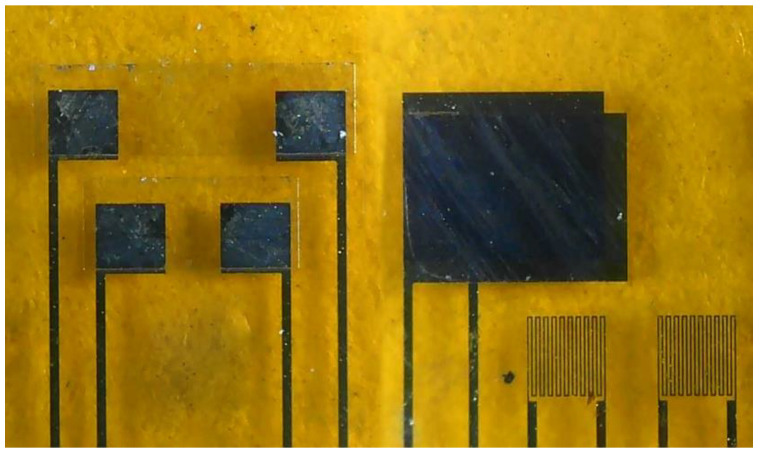
Optical microscope image of a flexible six-in-one microsensor.

LTC 9305 was then coated as the dielectric layer of a pressure sensor. It has the effects of high mechanical strength, corrosion resistance, electrochemical environment resistance, and stretchability. The completed schematic diagram is shown in Figure 2.

## 3. Correction of the Flexible Six-in-One Microsensor

Six physical quantities of the flexible six-in-one microsensor were corrected, including the voltage, current, electrical conductivity, temperature, flow, and pressure.

### 3.1. Micro-Voltage, Current, and Electrical Conductivity Correction of the Flexible Six-in-One Microsensor

A digital multimeter was used to check whether the measurements of the micro-voltage sensor and micro-current sensor were correct. The battery voltage and current were verified using an electric meter with the correction procedures. Afterwards, the micro-sensing head was connected to the battery. When there was no error, it could be verified as the normal operation of the micro voltage, current, and electrical conductivity sensors.

### 3.2. Temperature Correction of the Flexible Six-in-One Microsensor

**Figure 3:**
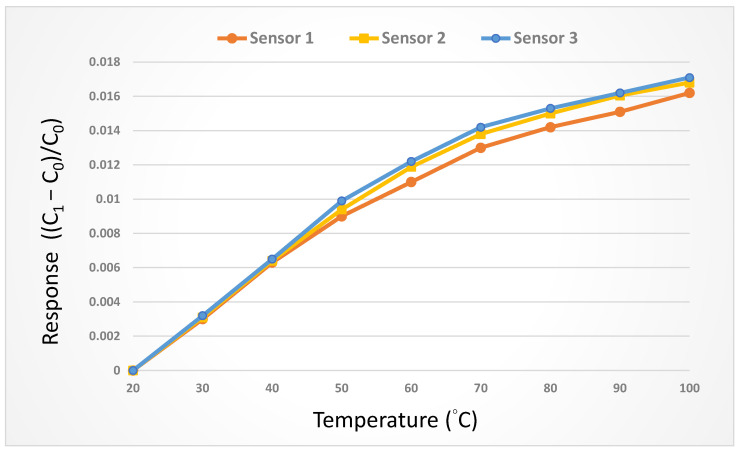
Correction curve of the micro-temperature sensor.

The micro-temperature sensor and the thermometer were placed in the DENG YNG DS45 Drying Oven. After the reference control temperature was stabilized, the resistivity of the micro-temperature sensor was extracted. In the working temperature range, the resistivity was extracted at intervals of 5 °C. The micro-temperature sensor was corrected three times, and the average value was taken to obtain the correction curve. In order to increase the accuracy, the three micro-temperature sensors were corrected three times and the average value was taken. The corrected curve is shown in Figure 3.

### 3.3. Flow Correction of the Flexible Six-in-One Microsensor

**Figure 4:**
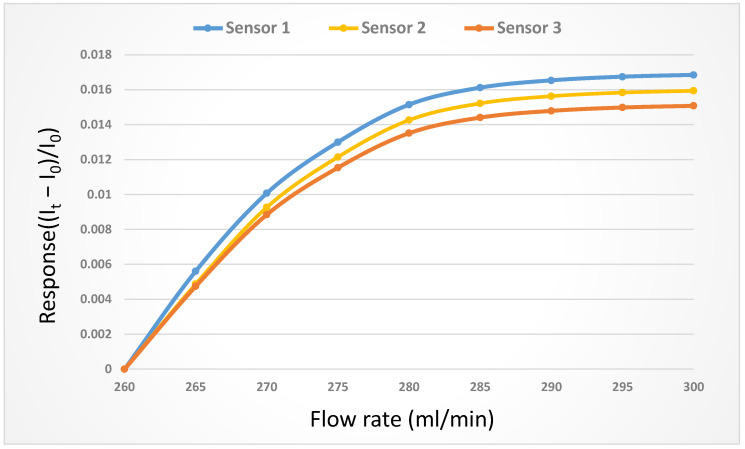
Correction curve of the micro-flow sensor.

The flow was corrected using a corrosion resistant liquid diaphragm pump, which provided a continuous and steady flow. The only component in the diaphragm pump which contacted the delivered liquid was the spherical valve, so that it could perform delivery without being damaged by the liquid. In industrial production, the diaphragm pump is mainly used to deliver the corrosive liquid; hence, it is a suitable pump of VRFBs for delivering vanadium electrolytes. The flow range of the liquid diaphragm pump (corrosion resistant) was 0~400 mL/min, while the flow accuracy was ±2%, and the correction curve is shown in Figure 4.

### 3.4. Pressure Correction of the Flexible Six-in-One Microsensor

**Figure 5:**
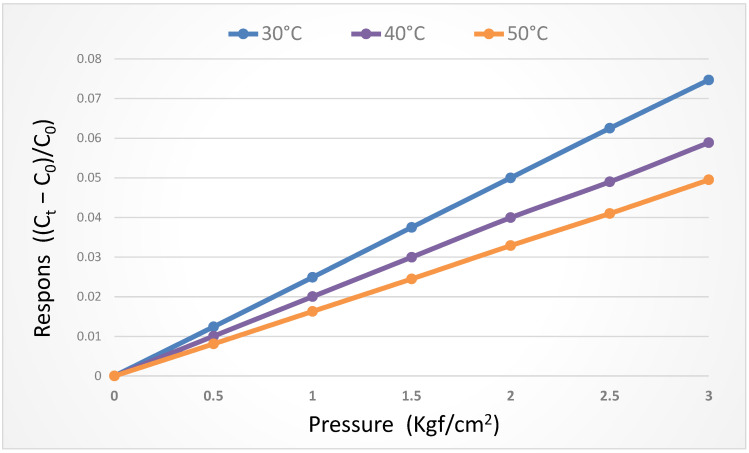
Dimensionless correction curve of the micro-pressure sensor.

For the micro-pressure sensor correction test, the micro-pressure sensor was corrected under the pressure of 0~3 bar in the operating condition when the temperature was fixed at 30 °C, 40 °C, and 50 °C, respectively. It was observed that the change in capacitance decreased gradually as the temperature rose. As the dielectric layer was made of PI polymer, the pressure was applied repeatedly at the initial stage of correction, and the capacitance value was stabilized after unloading. The micro-pressure sensor correction curve was obtained in the final stage, as shown in Figure 5.

## 4. Development of VRFB

**Figure 6:**
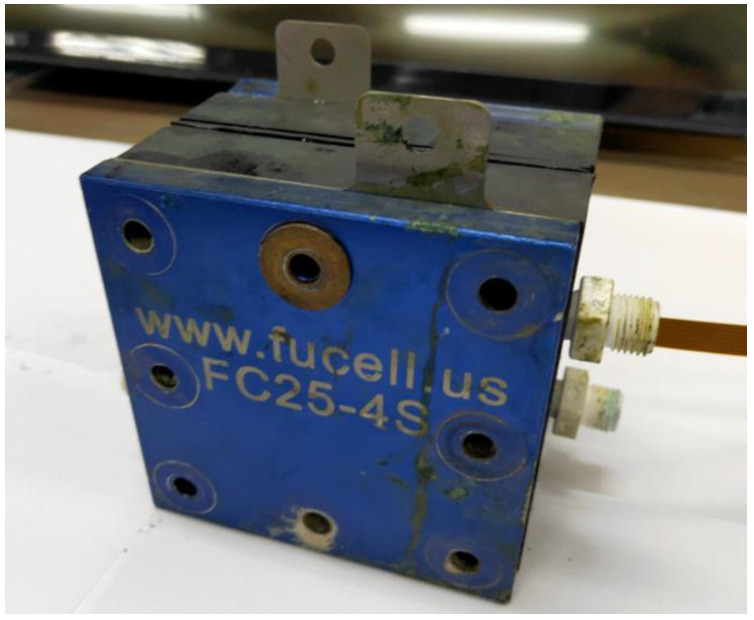
Vanadium redox flow battery.

**Figure 7:**
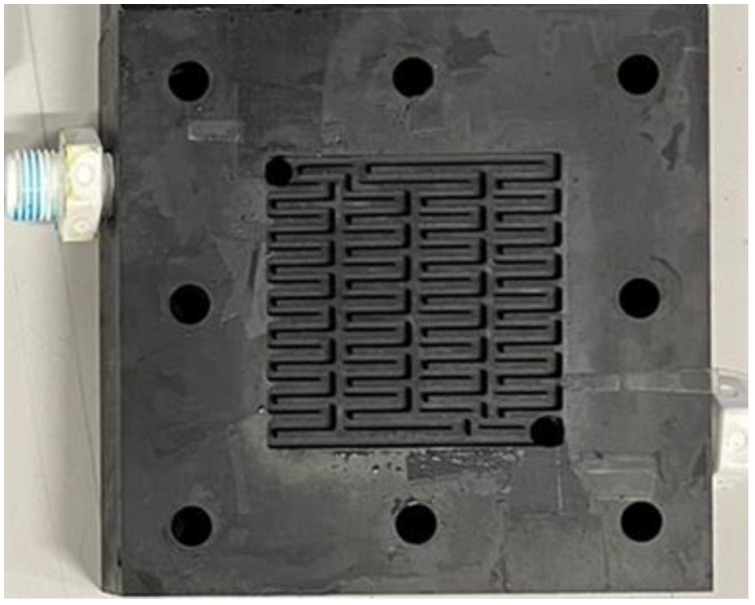
Four-serpentine nonparallel channel bipolar plate.

Figure 6 shows the vanadium redox flow battery. The serpentine flow field is the most familiar form of VRFB channel design, but its defect is that the longer the channel is, the higher the power that is consumed by the external pump. Therefore, to resolve the defect in the serpentine flow field, the bipolar plate flow field was deemed as the improved four-serpentine nonparallel flow field in this study, as shown in Figure 7. This improved four-serpentine nonparallel flow field could solve the problems related to, for example, a small electrolyte passing through the area of the parallel flow field, the overlong channel length of the serpentine flow field, and the high-power consumption of the pump.

**Figure 8:**
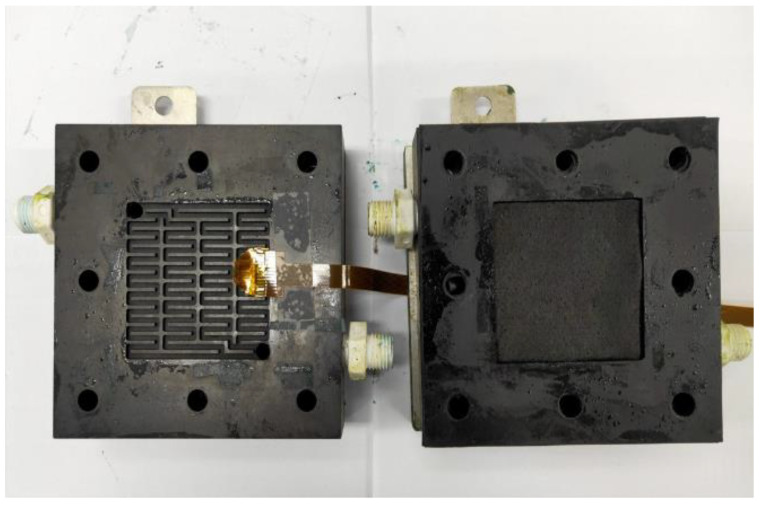
Flexible six-in-one micro-sensor embedded in the VRFB.

In this study, a 5 mm carbon felt was selected as the reaction center of the VRFB. The 5 mm carbon felt can absorb more electrolytes, so that the VRFB encompasses a larger quantity of chemical reaction. It must, at least have a low body impedance, good mechanical properties, compressibility, and low contact impedance. The proton exchange membrane used in this study is the F31-B175 proton exchange membrane. In order to prevent the leakage of vanadium electrolytes induced by the gap between the proton exchange membrane and the carbon felt, a 4 mm thick corrosion resistant rubber pressure equalizing pad was mounted on both sides of the membrane material, and a piece of 0.34 mm carbon paper was used in this study. The flexible six-in-one microsensor embedded in VRFB is shown in Figure 8. 

## 5. 200 H Long-Term Test

**Figure 9:**
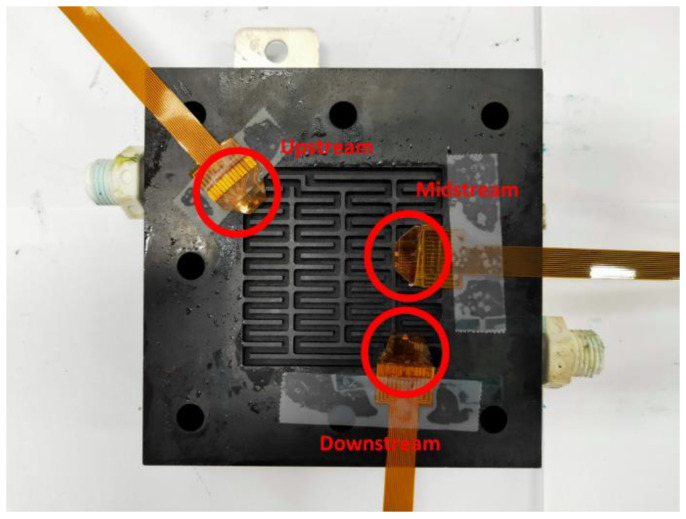
Flexible six-in-one miniature sensors embedded upstream, midstream, and downstream of the VRFB.

The flexible six-in-one microsensor was embedded in the VRFB for 200 h of long-term measurements in the upstream, midstream, and downstream of the internal flow channel (Figure 9).

**Figure 10:**
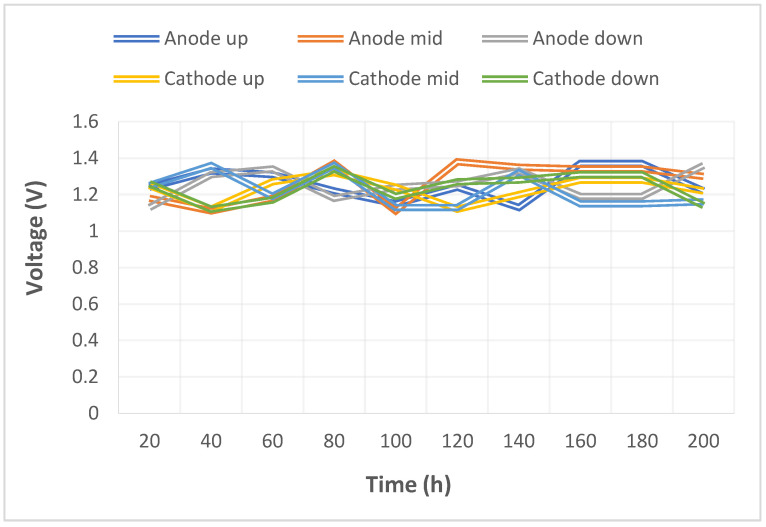
Long-term test curve of the micro-voltage sensor.

**Figure 11:**
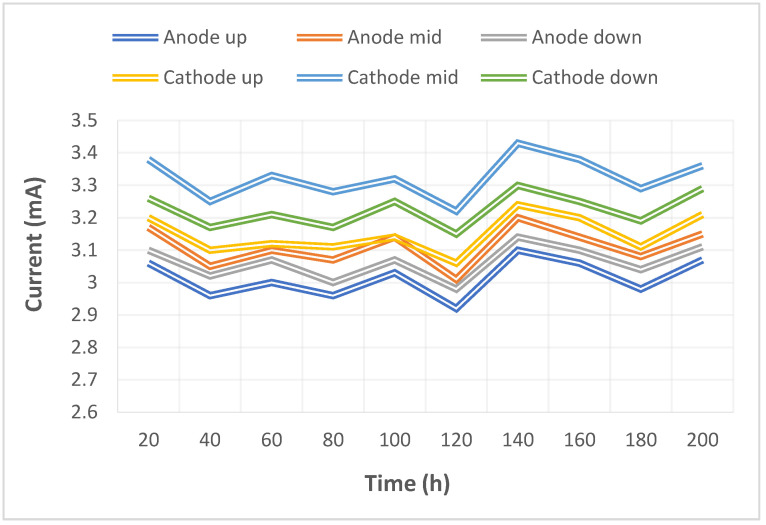
Long-term test curve of the micro-current sensor.

**Figure 12:**
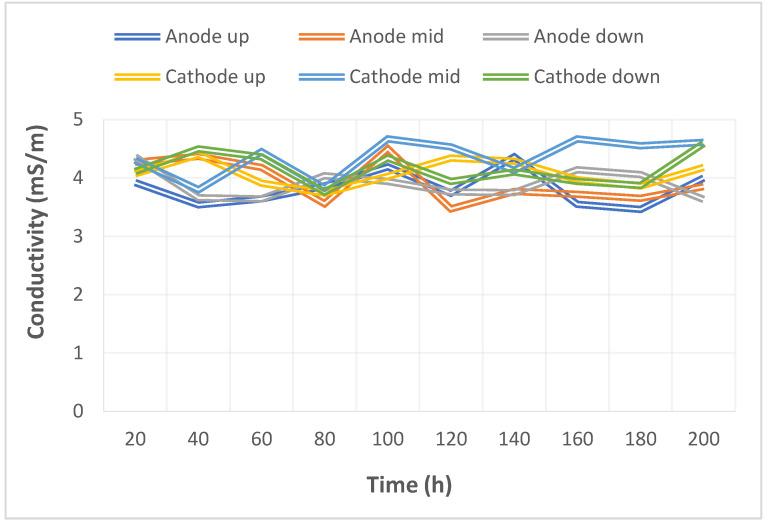
Long-term test curve of the micro-electrical conductivity sensor.

**Figure 13:**
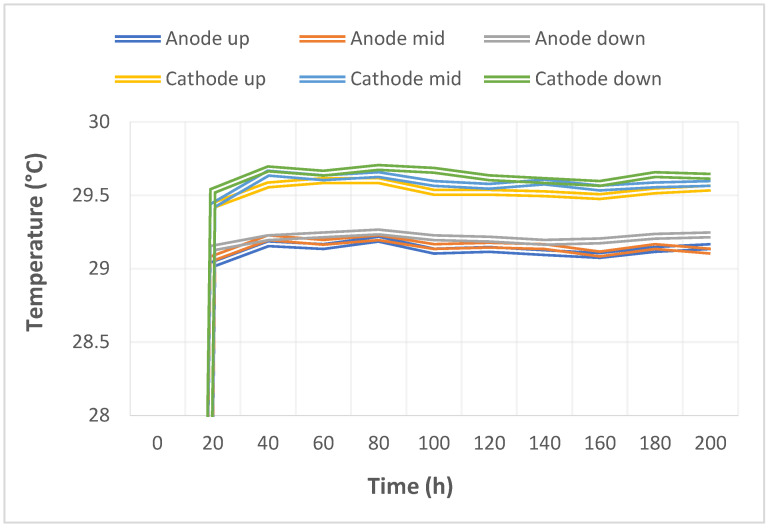
Long-term test curve of the micro-temperature sensor.

**Figure 14:**
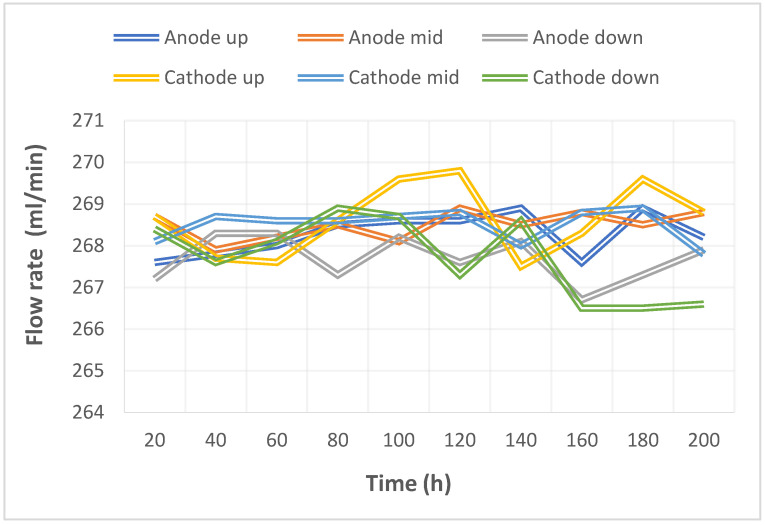
Long-term test curve of the micro-flow sensor.

**Figure 15:**
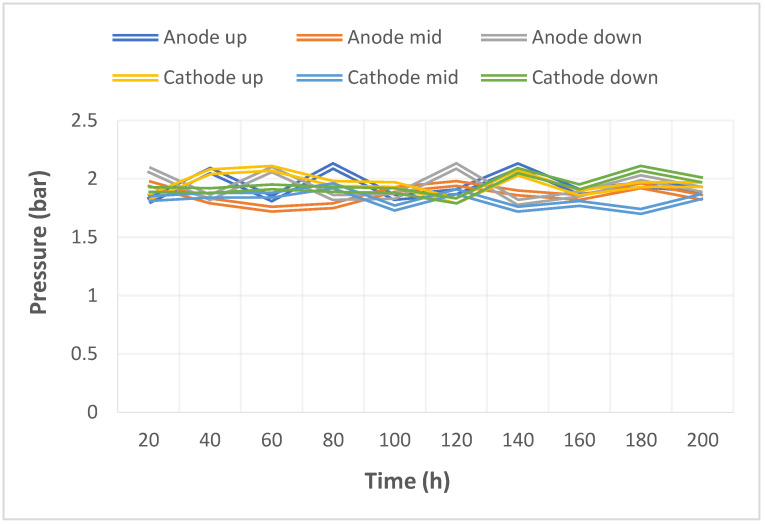
Long-term test curve of the micro-pressure sensor.

The pump used in this study could supply a constant flow at all times, so the upstream, midstream, and downstream voltages were stabilized at 1.1 V~1.4 V during charging, as shown in Figure 10. The distribution data of the current in the upstream, midstream, and downstream of the anodes, and in the upstream, midstream, and downstream of the cathodes are all displayed in Figure 11. It was observed that the reaction process was very stable, as the collector plate could steadily and uniformly lead the current in the bipolar plate and channel. The distribution data of electrical conductivity in the upstream, midstream, and downstream of the anodes, and in the upstream, midstream, and downstream of the cathodes are all shown in Figure 12. It was observed that the reaction process was also very stable because there were no significant changes observed between the voltage and current, and there were also no significant changes seen in the electrical conductivity. Figure 13 shows the 200 h internal temperature distribution of the VRFB. It was observed that the temperature had an obvious uptrend at the beginning, mainly due to the heat generated by the internal impedance. Therefore, the internal temperature of the battery also rose. However, it was found that the temperature of the cathode was a little higher than that of the anode by 0.5 °C on average, which was caused by the heat generated from cathode activation loss. A micro-flow sensor was used for observing the internal flow of the battery. If the flow change observed was large, the battery efficiency might therefore be reduced, and if the flowrate variation was apparent, whether there is foreign matter blocking inside could be detected in real time. Figure 14 shows the 200 h flow distribution in the upstream, midstream, and downstream of the anode and the cathode of the VRFB. It was observed that the flow was steady, meaning that the battery was able to work normally. A micro-pressure sensor was used for observing whether there was an obvious pressure variation inside the battery. This is because an obvious pressure variation indicates that the battery may either be blocked or leaky. The long-term test curve is shown in Figure 15. It can be observed that there was no obvious variation of pressure, meaning the battery was able to function normally.

## 6. Conclusions

In this study, when the microsensor process was changed to a lift-off process, the quality and the stability were both improved significantly. Previously, this research and development team used the wet etching process to produce microsensors [20]. The wet etching process is relatively inexpensive; however, it is difficult to control the quality stability. As it is not easy to control the time, over etching often occurs, resulting in larger errors of microsensors.

The flexible six-in-one microsensor utilizes micro-electro-mechanical systems technology to successfully integrate temperature, flow, voltage, and current on the PI substrate, and selects the PI material with chemical corrosion resistance and acid resistance as the protective layer. The flexible six-in-one microsensor has five characteristics: thin thickness, small structure area, high sensitivity, real-time measurement, and arbitrary arrangement. It can be embedded in the flow channel plate without affecting the sealing condition of the VRFB. The reliability test was performed after the flexible six-in-one microsensor was developed successfully. The microsensor was then embedded in the VRFB to perform a 200 h long-term charge-discharge test to instantly detect the electrical conductivity, voltage, current, temperature, electrolyte flow, and channel pressure distribution patterns in the activation to the degenerative processes of the VRFB.

## Data Availability

Not applicable.

## References

[1] Kumar A., Luthra S., Mangla S.K., Reyes J.A.G., Kazancoglu Y. (2023). Analysing the adoption barriers of low-carbon operations: A step forward for achieving net-zero emissions. Resour. Policy.

[2] Das A., Saini V., Parikh K., Parikh J., Ghosh P., Tot M. (2023). Pathways to net zero emissions for the Indian power sector. Energy Strategy Rev..

[3] Shakya S.R., Nakarmi A.M., Prajapati A., Pradhan B.B., Rajbhandari U.S., Rupakheti M., Lawrence M.G. (2023). Environmental, energy security, and energy equity (3E) benefits of net-zero emission strategy in a developing country: A case study of Nepal. Energy Rep..

[4] Bogdanov S., Pugach M., Parsegov S., Vlasov V., Ibanez F.M., Stevenson K.J., Vorobev P. (2023). Dynamic modeling of vanadium redox flow batteries: Practical approaches, their applications and limitations. J. Energy Storage.

[5] Anika O.C., Nnabuife S.G., Bello A., Okoroafor E.R., Kuang B., Villa R. (2022). Prospects of low and zero-carbon renewable fuels in 1.5-degree net zero emission actualisation by 2050: A critical review. Carbon Capture Sci. Technol..

[6] Khataee A., Nederstedt H., Jannasch P., Lindström R.W. (2023). Poly(arylene alkylene)s functionalized with perfluorosulfonic acid groups as proton exchange membranes for vanadium redox flow batteries. J. Membr. Sci..

[7] Díez E.S., Ventosa E., Guarnieri M., Trovò A., Flox C., Marcilla R., Soavi F., Mazur P., Aranzabe E., Ferret R. (2021). Redox flow batteries: Status and perspective towards sustainable stationary energy storage. J. Power Sources.

[8] Sharma J., Kulshrestha V. (2023). Advancements in polyelectrolyte membrane designs for vanadium redox flow battery (VRFB). Results Chem..

[9] Salmon N., Alcántara R.B. (2022). A global, spatially granular techno-economic analysis of offshore green ammonia production. J. Clean. Prod..

[10] Ren J., Li Y., Wang Z., Sun J., Yue Q., Fan X., Zhao T. (2023). Thermal issues of vanadium redox flow batteries. Int. J. Heat Mass Transf..

[11] Liu B., Tang C.W., Jiang H., Jia G., Zhao T. (2021). Carboxyl-functionalized TEMPO catholyte enabling high-cycling-stability and high-energy-density aqueous organic redox flow batteries. ACS Sustain. Chem. Eng..

[12] Liu B., Tang C.W., Sheong F.K., Jia G., Zhao T. (2022). Artificial bipolar redox-active molecule for symmetric nonaqueous redox flow batteries. ACS Sustain. Chem. Eng..

[13] Zhang B., Zhang X., Liu Q., Zhao M., Yang Z., Fu Y., Zhang E., Wang K., Wang G., Zhang Z. (2022). Swelling-induced ethylmorpholinium-functionalized adamantane-containing poly(aryl ether ketone) membranes with high conductivity and selectivity for vanadium redox flow batteries. J. Power Sources.

[14] Huang Z., Mu A., Wu L., Wang H. (2022). Vanadium redox flow batteries: Flow field design and flow rate optimization. J. Energy Storage.

[15] Charvát J., Mazúr P., Paidar M., Pocedič J., Vrána J., Mrlík J., Kosek J. (2021). The role of ion exchange membrane in vanadium oxygen fuel cell. J. Membr. Sci..

[16] Hu C., Dong Y., Zhang W., Zhang H., Zhou P., Xu H. (2023). Clean preparation of mixed trivalent and quadrivalent vanadium electrolyte for vanadium redox flow batteries by catalytic reduction with hydrogen. J. Power Sources.

[17] Jienkulsawad P., Jirabovornwisut T., Chen Y.S., Arpornwichanop A. (2023). Effect of battery material and operation on dynamic performance of a vanadium redox flow battery under electrolyte imbalance conditions. Energy.

[18] Tripathi R., Malgan P., Magdum P. (2021). Pressure drop analysis for hydraulic valves. Mater. Today Proc..

[19] Rao P., Gundlapalli R., Jayanti S. (2022). Assessment of hydrodynamic performance of vanadium redox flow batteries at low temperatures. J. Energy Storage.

[20] Lee C.Y., Lee S.J., Chen C.H., Hsu S.Y., Chien Y.H., Lee T.J. (2020). Flexible 4-in-1 microsensor for in-situ diagnosis of electric motorcycle fuel cell range extender. Sens. Actuators A Phys..

